# Surgery for thoracoabdominal aortic aneurysm

**DOI:** 10.1186/1749-8090-8-S1-O28

**Published:** 2013-09-11

**Authors:** Z Mitrev, T Anguseva, N Hristov, E Stoicovski, E Idski

**Affiliations:** 1 Special Hospital for Surgery Fillip II, Skopje, Macedonia

## Background

Repair of thoracoabdominal aortic aneurysms (TAAAs) is combined with hemorrhagic shock, cardiac arrest and multisystem organ failures like a most frequent causes of death, and paraplegia and renal failure are most devastating complications.

## Material and methods

From January 2008 till May 2013, 16 patients age 55 ± 7 years, had been treated. They were symptomatic, with mean aneurysm dimension of 10 ± 2 centimeters. One patient had Crawford type IV TAAA that was unrecognized, mistaken for tumor and biopsied following explorative laparotomy for acute abdominal pain in another hospital. Surgery was performed through thoracophrenolaparotomy, with construction of composite end to end prosthesis between tubular and bifurcated graft and proximal end to side prosthesis implantation on thoracic aorta. End to end anastomosis with both iliac arteries was performed, followed by implantation of the celiac trunk, superior mesenteric and renal arteries over short 10 mm vascular grafts on the prosthesis.

## Results

There were no operative deaths. All patients remained hemodinamically stable throughout the procedure. 7 patients had minimal blood loss postoperatively and were extubated within first 24 hours. They had uneventful postoperative stay and were released home between 8-10th postoperative day. 4pts required prolonged ventilation. Patient who had previous laparotomy developed infection of the surgical incision that required surgical reintervention. Patient who was reoperated for arch aneurysm had prolonged ventilation, tracheostomy, intestinal bleeding, sepsis and paraplegia (died after 7 months). Mortality rate was 12,5% (2pts).

## Conclusion

Without usage of extracorporal circulation and reduced ischemic time during TAA repair, this technique avoids the inevitable operative complications encountered with hypothermic circulatory arrest, partial cardiopulmonary bypass, partial left heart bypass, or clamp-and-sew strategy.

